# Gate-controlled electron quantum interference logic

**DOI:** 10.1039/d2nr04423d

**Published:** 2022-09-07

**Authors:** Josef Weinbub, Mauro Ballicchia, Mihail Nedjalkov

**Affiliations:** Christian Doppler Laboratory for High Performance TCAD, Institute for Microelectronics TU Wien Austria josef.weinbub@tuwien.ac.at; Institute for Microelectronics TU Wien Austria

## Abstract

Inspired by using the wave nature of electrons for electron quantum optics, we propose a new type of electron quantum interference structure, where single-electron waves are coherently injected into a gate-controlled, two-dimensional waveguide and exit through one or more output channels. The gate-controlled interference effects lead to specific current levels in the output channels, which can be used to realize logic gate operations, *e.g.*, NAND or NOR gates. The operating principle is shown by coherent, dynamic Wigner quantum electron transport simulations. A discussion of classical simulations (Boltzmann) allows to outline the underlying process of interference. Contrary to other electron control approaches used for advanced information processing, no magnetic or photonic mechanisms are involved.

## Abstract 2

We pursue the idea of gate-controlled interference phenomena of electron waves and show potential use cases for logic applications.

## Graphical abstract

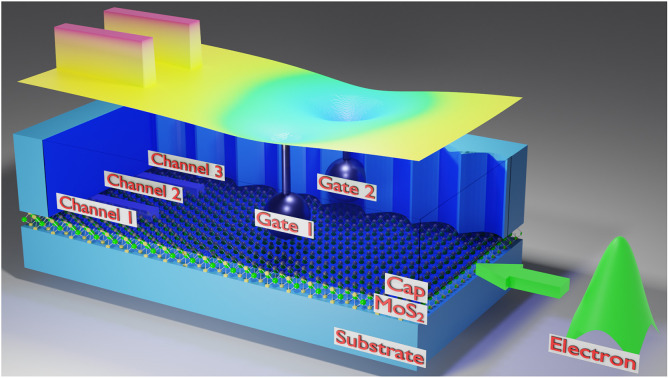

By treating electrons as waves, electron quantum optics provides the basis for conducting quantum optics-like investigations in a fermionic picture.^1–3^ Compared to quantum optics, solid-state electron approaches have the advantage in terms of size, scalability, and the ability of the charge degree of freedom to be easily measurable, with the drawback of a much shorter coherence time. Key enabling technologies are: electron sources (*e.g.*, tunable quantum dot designs in Si^4^ and MoS_2_ ^5^ and two-electron sources^6^), coherent circuits,^7^ and electron detection.^8^ All these technologies are key to electronic quantum applications in sensing,^9^ metrology,^10^ tomography,^11^ and information processing (*i.e.*, flying charge qubit systems^1,12^). As is shown in this work, electron quantum optics also provides particular exciting opportunities for a novel type of quantum interference structure. Applications of interference effects have a long history, ranging from superconductor,^13–16^ single-atom,^17^ and molecular junction^18–20^ approaches to early investigations into logic devices.^21^ In contrast, here we propose to use a different operational principle: electron interference. The latter is characterized with a sensitivity to geometry, potential values, material parameters, and other physical attributes. In our study, we focus on a particular two-dimensional (2D) waveguide design, inspired by astonishing advances in 2D materials research in recent years.^22,23^ As expected, our simulations confirm that the manifesting electron density interference patterns are entirely governed by quantum effects involved in the electron evolution. Effects of tunneling, non-locality, and boundary reflection are identified. Comparative simulations of the classical evolution show a very different picture (see Appendix A) which underlines that the main phenomena responsible for the logic operation is interference.

Fig. 1 shows a schematic representation of the suggested interference logic structure. For the 2D waveguide we consider a 22 × 40 nm^2^ molybdenum disulfide (MoS_2_) single-layer; a 2D semiconductor of the family of transition metal dichalcogenides (TMDCs). Single-layer MoS_2_ is a direct-bandgap (1.8 eV) semiconductor, which makes it well-suited for low-power applications.^24^ First principles band structure calculations showed that for n-type single-layer MoS_2_ the K, K′ valleys, which are well-separated in energy from the satellite valleys, are most relevant for low-field transport. A parabolic band with electron mass *m* = 0.48*m*_0_ (with *m*_0_ being the mass of the free electron) gives a perfect description of the conduction band valley around the K point and was thus used as effective mass in our modeling. The choice of MoS_2_ is by way of example and rooted in the fact that this particular material found widespread research attention in recent years^22,25^ and particularly concerning single-electron sources.^5^ However, other interesting materials (offering attractive ballistic properties) for the here considered transport layer are available, such as (bilayer) graphene (see, for instance,^26^ and references therein).

**Fig. 1:**
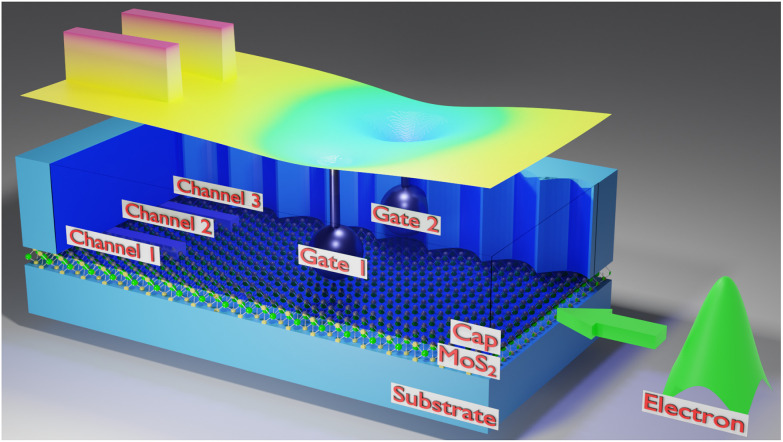
Schematic representation of the interference logic structure. An exemplary potential energy distribution of the waveguide derived from biased gates and the structure is shown on top. The blue cap layer is partially made transparent to show the inside.

The waveguide is insulated by a cap and a substrate layer on the top and bottom, respectively, and offers reflective boundaries along *x* = 0 nm and *x* = 22 nm (Fig. 2 and 3). The gates are embedded in the cap layer: the metal gates are solid spheres with a radius of 2 nm positioned 3 nm (relative to the sphere center) above the waveguide, resulting in 1 nm cap oxide material (calcium fluoride^27^) between the surface of the gates and the MoS_2_. The charge and shape of the gates and the distance between them, the transport domain geometry, the output channel walls (potential energy of 0.1 eV and dimensions 2 × 10 nm^2^ centered at *x* = 7 nm and 15 nm, respectively, and ranging from *y* = 30–40 nm), and the boundaries, determine the corresponding potential energy distribution in the channel (see an exemplary potential energy distribution overlaid in Fig. 1).

**Fig. 2:**
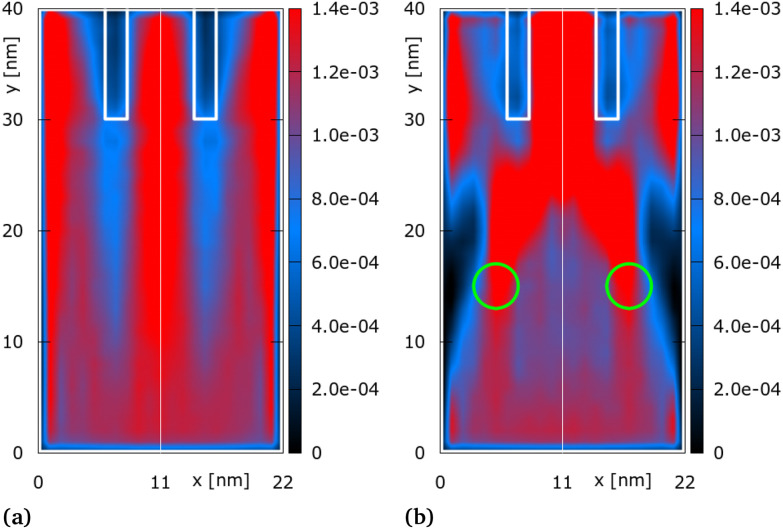
Distribution of quantum electron density [a.u.] for two symmetric gate configurations: *ϕ*_*G*1_ = *ϕ*_*G*2_ = 0 V (a) and *ϕ*_*G*1_ = *ϕ*_*G*2_ = 0.21 V (b). The output channel walls are shown in white. Green circles indicate location of gates.

**Fig. 3:**
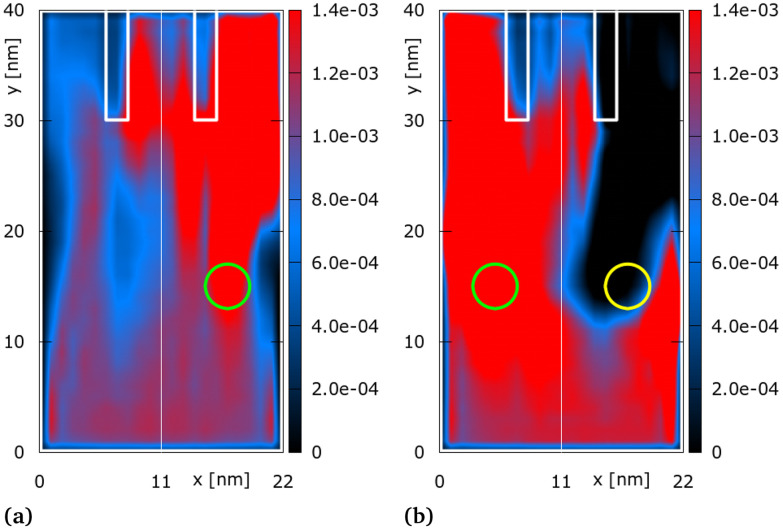
Distribution of quantum electron density [a.u.] for two asymmetric gate configurations: *ϕ*_*G*1_ = 0V and *ϕ*_*G*2_ = 0.21 V (a) and *ϕ*_*G*1_ = 0.21 V and *ϕ*_*G*2_ = −0.21 V (b). The yellow circle indicates the location of the negatively charged gate.

Electrons with a mean momentum of 0.94 nm^−1^ enter on one side of the structure and cross it within approximately 200 fs. The injection frequency of today's single-electron sources can reach 10 GHz(ref. 4) (which is in the order of other state-of-the-art approaches^28^), which translates to a 100 ps injection period, being orders of magnitudes slower than the electron crossing time. Therefore, the injected electrons can be well considered as non-interacting. Moreover, the current and general performance of the interference structure is thus primarily determined by the necessary upstream single-electron source.

The potential energy distribution *V* = −*qϕ* (*ϕ* being the electric potential and *q* the electron charge, so that a negative or positive gate bias represents a repulsive barrier or an attractive well for the electrons, respectively) together with the physical characteristics of the injected electrons, determine the electron evolution in the 2D waveguide. Stochastic evolution simulations (see recent monographs^29,30^ and reviews^3,31^)are used for various gate configurations to calculate the electron and current densities and the total current levels in the output channels.

Regarding the theoretical framework for the simulation backend (for a recent review of computational methods for quantum electron transport and structure simulations see ref. 3): the quantum mechanics of the Schrödinger equation is reformulated by the Wigner (or the equivalent density matrix) formalism in phase space. The latter involves quantum statistics and introduces a function which is the quantum counterpart of the Boltzmann distribution function.^30^ This is the formalism of choice in our research. It offers a rigorous quantum description of coherence processes, allows to account for an interaction with the lattice (*e.g.*, thermal effects due to phonon scattering), and ensures a seamless transition to a classical description in regions with classical behavior. More concretely, we apply the signed-particle model to the Wigner transport equation.^32^ This model extends the conventional, classical particle model applied in the Boltzmann theory by assigning quantum information to the particles. Quantum particles evolve in a classical way, however, they carry a plus or minus sign, are generated by the Wigner potential *V*_W_, and annihilate each other if they occupy the same point in phase space. Ultimately, we solve the Wigner transport equation by a signed-particle stochastic approach provided by our ensemble Monte Carlo solver ViennaWD.††http://www.iue.tuwien.ac.at/software/viennawd/^33^ As was mentioned before, we focus on the coherent case to clearly highlight the manifesting quantum effects which anyway control the electron evolution in this considered ballistic structure. However, we do compare to classic transport (Boltzmann) to identify the differences (see Appendix A).

The classical limit of the Wigner equation is obtained in the case of slowly varying potentials, giving rise to the ballistic Boltzmann equation.^32^ Accordingly, the signed-particle model reduces to the Boltzmann model and the very intuitive picture of classically evolving particles. This reduction provides a very convenient way to outline and analyze quantum effects in the electron evolution. An injected electron is assumed *free* and thus modelled as a minimum uncertainty wave packet, having a corresponding Wigner state^34^1
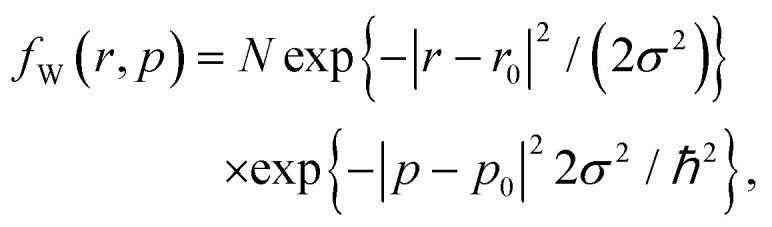
where *N* is a normalization constant. State (1) is characterized by a Gaussian distribution^35^ in any of the involved dimensions *x* and *y*.

The choice of the standard spatial deviation *σ* is a compromise between the spread of *f*_W_ in the position and momentum subspaces, respectively. The chosen *σ* = 16 nm ensures a small spreading of the involved frequencies, while still being in accordance with the waveguide dimensions: the value corresponds to a momentum deviation smaller than 0.1 nm^−1^ around *p*_0_ = 0.94 nm^−1^. This is around half of the maximal momentum along *y*-direction and corresponds to a kinetic energy of 71 meV of the injected electron and to an uncertainty of about 5 meV. The structure's dimensions and the involved energies imply a fundamental quantum process of transport.

The simulation setup conceptually resembles a double-slit experiment, where single electrons are shot consecutively towards a screen, until a stationary (steady-state) interference pattern is obtained.^36,37^ The probabilistic interpretation is of equivalent independent (uncorrelated) experiments aiming at accumulating sufficient statistics. However, in contrast to double-slit experiments, where a single barrier offers two slits, here, similar interference effects manifest by utilizing two potential energy wells in a waveguide.^36^

Fig. 2a considers no applied bias to the gates, Gate 1 (*G*_1_) and Gate 2 (*G*_2_). Electrons are injected in *y*-direction at the bottom (see green wave-packet in Fig. 1). The classical transport interpretation (see Appendix A) for this configuration is very clear and applies to all configurations discussed in the following: electrons move freely and are only reflected by the boundaries. They *feel* the two wall potentials only when entering into a direct contact with them and are reflected or continue the movement with a reduced speed depending on their kinetic energy. Thus, classical electrons firmly surround the walls. On the contrary, the quantum density shown in Fig. 2a is very different: a reduced density (blue regions) is shown not only surrounding the walls but also below the walls between *y* = 10 nm and *y* = 30 nm. The two wall potentials evoke a strong non-local action which shows the quantum character of the electron evolution. The region *y* < 10 nm is particularly interesting: for reference, the classical density is dominated by the injected distribution (see Appendix A). The quantum density instead shows a fine oscillating structure, marked by low density *scars*, which suggests interference effects in the quantum dynamics. The electron density is thus equally split into the three output channels, following the symmetry of the walls.

Fig. 2b shows the electron density for a symmetric gate bias configuration, *i.e.*, *ϕ*_*G*1_ = *ϕ*_*G*2_ = 0.21 V. The resulting peak of the superimposed electric potential of the two gates, as determined by the gate and structure geometry, is 0.17 V on the MoS_2_ surface, *i.e.*, both gates act as *attractive* wells. As a result, a focusing effect materializes: the majority of the electron density is guided towards output channel 2 (the central channel).

Here, we observe further elemental quantum phenomena: tunneling and penetration into the channel walls. Indeed, the potential barriers of the walls are higher than the central kinetic energy of the injected electrons (Gaussian distribution). According to classical (Boltzmann) evolution rules, only a few particles from the tail of the Gaussian can overcome the barrier. Besides, the classical action of the potential is local, the particles will move uniformly in the wall region, thus forming an even, low-density distribution. In contrast, the distribution in the walls is not even, while the density is comparable to the counterpart in the adjacent area. Thus the density in channels 1 and 3 has a contribution due to tunneling.

Fig. 3a shows the electron density for an asymmetric gate bias configuration, *i.e.*, *ϕ*_*G*1_ = 0 V and *ϕ*_*G*2_ = 0.21 V, corresponding to a resulting peak superimposed electric potential on the MoS_2_ surface of 0.14 V, *i.e.*, *ϕ*_*G*2_ acts as an *attractive* well in front of output channel 3 (right channel): the majority of the electron density is thus guided towards channel 3, less so towards channel 2, and considerably less to channel 1 (left channel). Fig. 3b shows the electron density for another representative asymmetric gate configuration where the gates are biased in an opposing manner, *i.e.*, *ϕ*_*G*1_ = 0.21 V and *ϕ*_*G*2_ = −0.21 V. This configuration corresponds to a resulting peak superimposed electric potential on the MoS_2_ surface of 0.11 V in front of channel 1, and a repulsive barrier of −0.11 V in front of channel 3. In this case the electron density guided towards channel 3 is almost completely blocked and the majority of the electron moves towards channel 1; a considerably smaller part of the electron density enters channel 2.

In the next section, we show how the gate-controlled interference in the depicted structure could be used for realizing logic gates. All geometry and electric potential details determine the interference behavior, which in the spirit of the above considerations cannot be reproduced after a simple scaling of dimensions and potentials. However, as long as the elemental quantum phenomena determine the transport process, we expect that their interplay will allow for defining logical gates, but of course with different controlling potentials and current levels.

Fig. 4 shows the calculated quantum currents for a representative selection of *ϕ*_*G*1_ biases relative to fixed *ϕ*_*G*2_ biases in the three output channels. The reported currents are directly linked to the injection period of the considered single-electron source. The period has been empirically chosen so as to allow for an optimal visualization of the electron densities rather than to, *e.g.*, minimize the current for reduced power consumption (a typical engineering-focus), which is beyond the scope of this work. The current at the output of each channel has been calculated by integrating the current density along the corresponding output cross-section. In turn, the current density is calculated from the first-order moment of the electron distribution function. Fig. 4a considers the case of *ϕ*_*G*2_ = 0 V; *G*_2_ is thus not active (see a similar scenario in Fig. 3b albeit for *G*_1_). For negative *ϕ*_*G*1_ biases, *ϕ*_*G*1_ repulses the majority of the current to channel 3, with a similar inverted behavior for positive biases with respect to channel 1. In case of *ϕ*_*G*1_ = 0 V, the electron is equally distributed between all three output channels as expected from symmetry considerations, which corresponds to the scenario depicted in Fig. 2a. The negative and positive *ϕ*_*G*1_ bias ranges *seem to offer a mirrored* behavior, however, both ranges correspond to entirely different physical scenarios of repulsion and attraction, leading to different interactions between the electrons and the potential energy landscape and thus to different current levels. Therefore, a fully-mirrored behavior is not to be expected. Fig. 4b considers the case of *ϕ*_*G*2_ = 0.105 V. The negative bias range offers similar general behavior as for *ϕ*_*G*2_ = 0 V (see Fig. 4a), however, for the symmetric case *ϕ*_*G*1_ = *ϕ*_*G*2_ = 0.105 V a focusing effect with respect to channel 2 manifests (similar to Fig. 2b). A further increased *ϕ*_*G*1_ bias results in a deeper well potential at *G*_1_ over *G*_2_, thus the majority of the current manifests in channel 1. Fig. 4c considers the case of *ϕ*_*G*2_ = 0.21 V; *G*_2_ acts as an even stronger attractive well. Yet another intriguing example of quantum non-locality is seen here for negative biases: although *ϕ*_*G*2_ has doubled (compare with Fig. 4b), the current in channel 1 and 2 stays roughly the same but in channel 3 (located behind *G*_2_) is further reduced, demonstrating the wave nature of the evolution.

**Fig. 4:**
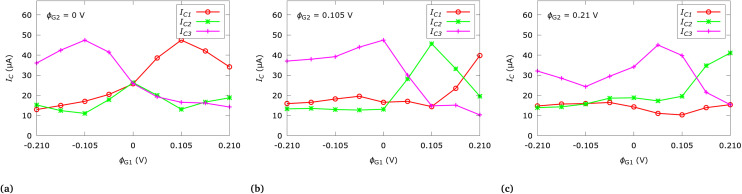
Output channel quantum currents *I*_*C*1,2,3_ for a set of gate biases *ϕ*_*G*1,2_.

Table 1 provides an extended overview of the gate bias to channel current relations and together with Table 2 shows various exemplary interpretations as logic gates: particularly important are *universal* NAND and NOR gates as both are *functionally complete* and as such can be used to implement all possible Boolean functions.^38^ In conventional logic, a NAND gate is defined by the *truth* table 00 → 1, 01 → 1, 10 → 1, 11 → 0 and the NOR gate by 00 → 1, 01 → 0, 10 → 0, 11 → 0, mapping two input states to one output state for the four binary-based configurations. In a similar fashion, additional logic gates can be identified, *e.g.*, AND and NOT gates.

**Table 1:** Output channel quantum currents *I*_C1,2,3_ (μA) for an extended set of gate biases *ϕ*_G1,2_ (V) relative to Fig. 4. The GATE column shows individual rows of the truth tables of realizable NAND and NOR gates according to the rules shown in Table 2; framed current values highlight the utilized channel to realize the logic

*ϕ*_G1_	*ϕ*_G2_	*I*_C1_	*I*_C2_	*I*_C3_	GATE
−0.21	−0.21	9	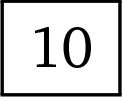	9	NOR: 11 → 0
−0.21	−0.105	10	14	28	
−0.21	0	13	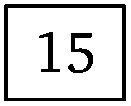	36	NOR: 10 → 0
−0.21	0.105	16	13	37	
−0.21	0.21	15	14	32	
−0.105	−0.21	28	14	10	
−0.105	−0.105	19	31	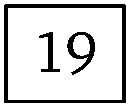	NAND: 00 → 1
−0.105	0	17	11	47	
−0.105	0.105	18	13	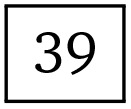	NAND: 01 → 1
−0.105	0.21	16	16	24	
0	−0.21	36	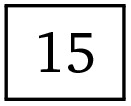	13	NOR: 01 → 0
0	−0.105	47	11	17	
0	0	26	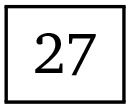	26	NOR: 00 → 1
0	0.105	17	13	47	
0	0.21	14	20	34	
0.105	−0.21	37	13	16	
0.105	−0.105	39	13	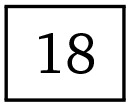	NAND: 10 → 1
0.105	0	47	13	17	
0.105	0.105	15	46	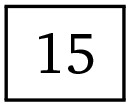	NAND: 11 → 0
0.105	0.21	10	20	40	
0.21	−0.21	32	14	15	
0.21	−0.105	24	16	16	
0.21	0	34	20	14	
0.21	0.105	40	20	10	
0.21	0.21	15	41	15	

**Table 2:** Exemplary logic gate definitions based on *ϕ*_G_ and *I*_C_ values of Table 1: IN associates specific *ϕ*_G1,2_ (V) biases with input states 0 and 1; *C* is the output channel number; OUT associates specific *I*_*C*1,2,3_ thresholds (μA) with output states 0 and 1; and GATE shows the realizable logic gate

IN		*C*	OUT		GATE
0	1		0	1	
0	−0.21	2	≤20	>20	NOR
−0.105	0.105	3	≤16	>16	NAND

The introduced gate-controlled interference structure provides a new perspective towards advanced non-magnetic, low-power, and high performance classically operating logic. The electronic nature provides the opportunity for co-integration with conventional electronics. Furthermore, various adaptations are perceivable, for instance, additional gates, channels, or bias configurations would allow to introduce even more current states. Alternatively, the output channels can be arranged in a non-symmetric manner or can be merged outside of the structure to allow for further superpositioned states. Robustness is tuneable by increasing the current ranges. The shown design is in principle adaptable to other transport materials (*e.g.*, graphene), offering additional options for practical realizations. In addition to classical binary logic, the manifestation of several current levels at the individual output channels provides a path towards multi-valued logic, which, although having inherent higher robustness challenges,^38^ promises higher integration densities.^39^ The fact that different gate bias configurations allow for different current levels allows for reconfiguration: the same structure configuration can realize different logic gates. In addition, we restricted the here presented initial studies to utilizing a single output channel for realizing a particular logic gate. However, the fact that additional, specific current levels are available also in the remaining output channels provides a path towards parallelization as various logic operations can be potentially realized in parallel.

## Conflicts of interest

There are no conflicts to declare.

## Supplementary Material

## Figures and Tables

**Fig. 5:**
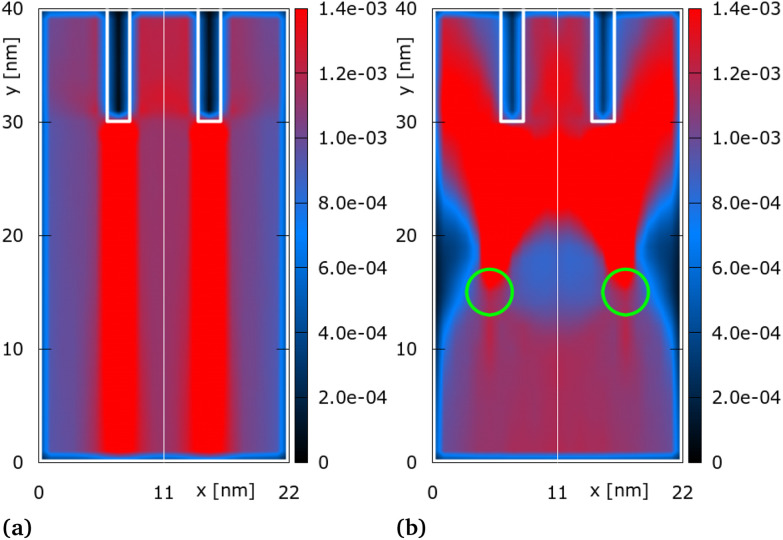
Distribution of classical electron density [a.u.] corresponding to the configurations shown in Fig. 2.

**Fig. 6:**
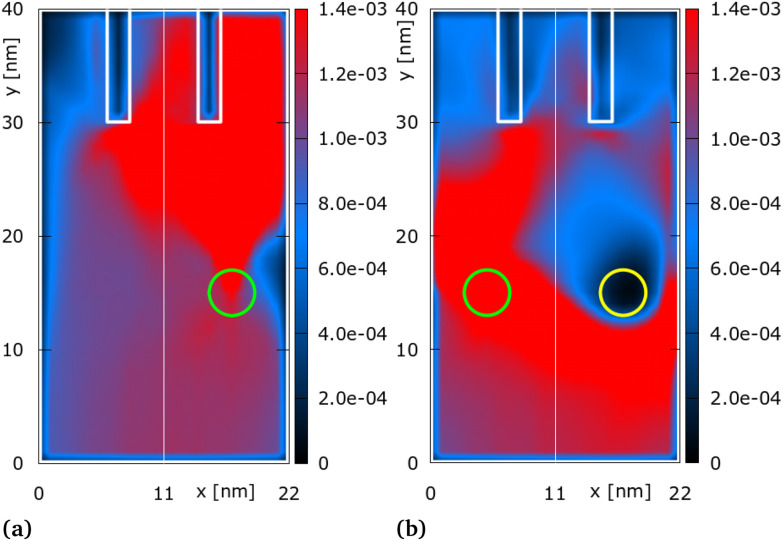
Distribution of classical electron density [a.u.] corresponding to the configurations shown in Fig. 3.

## Appendices

### Appendix: Classical Boltzmann considerations

There is a remarkable contrast between the principles of classical and quantum evolution. In particular, considering particle transport, classical evolution has a local character: point-like particles are accelerated over Newtonian trajectories by the local gradient of the potential: the electric field. The evolution can be analyzed in terms of probabilities (which in the case of independent processes sum up in an additive way). In particular the Gaussian distribution of an injected electron is interpreted as the probability to find the electron at a given position and momentum at a given time. Conversely, in the quantum case the Gaussian represents the single-electron state, which obeys the Heisenberg position-momentum uncertainty principle. The evolution is governed by the entire potential which imposes a non-local action on the also non-local electron state. The evolution is analyzed in terms of probability amplitudes which sum up to give the interference effects. These fundamental notions of non-locality, interference, and tunneling can interplay in a complicated way, already in the here discussed case of two gate potentials. This is in line with previous findings: In a related previous study of multiple impurity potential systems similar effects were shown: “*diffraction and interference effects, resonant flow regions, trapped and moving vortex flows, complex multiple scattering on several impurities, localisation effects and strong channel blocking*”.^40^

The classical Boltzmann evolution gives rise to fundamentally different electron density distributions (Fig. 5 and 6) as compared to the quantum counterparts shown in Fig. 2 and 3. Furthermore, the current is also very different and is approximately (15–51%) larger in the quantum case. For instance, a strong difference is observed when both gates are set to 0.21 V, where *I*_*C*2_ = 41 μA against a classical current of 20 μA. However, the classical results have to be taken with a grain of salt as in fact the here considered ballistic structure will enforce quantum transport effects.
